# Early expressions of psychopathology and risk associated with trans-diagnostic transition to mood and psychotic disorders in adolescents and young adults

**DOI:** 10.1371/journal.pone.0252550

**Published:** 2021-06-04

**Authors:** Jan Scott, Jacob J. Crouse, Nicholas Ho, Frank Iorfino, Nicholas Martin, Richard Parker, John McGrath, Nathan A. Gillespie, Sarah Medland, Ian B. Hickie

**Affiliations:** 1 Brain and Mind Centre, The University of Sydney, Sydney, Australia; 2 Institute of Neuroscience, Newcastle University, Newcastle, United Kingdom; 3 QIMR Berghofer Institute of Medical Research, Brisbane, Australia; 4 Virginia Institute for Psychiatric and Behavioral Genetics, Virginia Commonwealth University, Richmond, VA, United States of America; 5 Institute of Molecular Bioscience, The University of Queensland, Brisbane, Australia; Department of Psychiatry and Neuropsychology, Maastricht University Medical Center, NETHERLANDS

## Abstract

**Objectives:**

The heterogeneity and comorbidity of major mental disorders presenting in adolescents and young adults has fostered calls for trans-diagnostic research. This study examines early expressions of psychopathology and risk and trans-diagnostic caseness in a community cohort of twins and non-twin siblings.

**Methods:**

Using data from the Brisbane Longitudinal Twin Study, we estimated median number of self-rated psychiatric symptoms, prevalence of subthreshold syndromes, family history of mood and/or psychotic disorders, and likelihood of subsequent trans-diagnostic caseness (individuals meeting diagnostic criteria for mood and/or psychotic syndromes). Next, we used cross-validated Chi-Square Automatic Interaction Detector (CHAID) analyses to identify the nature and relative importance of individual self-rated symptoms that predicted trans-diagnostic caseness. We examined the positive and negative predictive values (PPV; NPV) and accuracy of all classifications (Area under the Curve and 95% confidence intervals: AUC; 95% CI).

**Results:**

Of 1815 participants (Female 1050, 58%; mean age 26.40), more than one in four met caseness criteria for a mood and/or psychotic disorder. Examination of individual factors indicated that the AUC was highest for subthreshold syndromes, followed by family history then self-rated psychiatric symptoms, and that NPV always exceeded PPV for caseness. In contrast, the CHAID analysis (adjusted for age, sex, twin status) generated a classification tree comprising six trans-diagnostic symptoms. Whilst the contribution of two symptoms (need for sleep; physical activity) to the model was more difficult to interpret, CHAID analysis indicated that four self-rated symptoms (sadness; feeling overwhelmed; impaired concentration; paranoia) offered the best discrimination between cases and non-cases. These four symptoms showed different associations with family history status.

**Conclusions:**

The findings need replication in independent cohorts. However, the use of CHAID might provide a means of identifying specific subsets of trans-diagnostic symptoms representing clinical phenotypes that predict transition to caseness in individuals at risk of onset of major mental disorders.

## Introduction

Globally, unipolar, bipolar, and psychotic disorders are ranked as three of the four most burdensome conditions in individuals aged less than 25 [1]. The first onset of full-threshold episodes of each of these disorder occurs before the age of 30 in 75% cases [1, 2]. However, it is argued that the high prevalence of longitudinal and concurrent co-occurrences of mood and psychotic symptoms and disorders means that comorbidity is the rule rather than the exception in adolescents and young adults [2–6]. This observation is supported by recent research including studies based on Danish registers (~6 million persons) and World Mental Health Surveys (~146,000 respondents from 27 countries) which demonstrate that (a) comorbidity within mental disorders is pervasive, and (b) those with onsets in late adolescence and/or early adulthood have an increased risk of developing other mental disorders over the following 15 years [3, 4]. The complexity and heterogeneity of the evolution of mental disorders presenting during adolescence and early adulthood (which is the peak age range for onset of adult-pattern psychiatric conditions) have exposed significant concerns regarding the reliability, validity and applicability of traditional diagnostic categories [5, 6]. As such, many experts now advocate the use of trans-diagnostic staging models as a more constructive strategy for research, prevention, and clinical treatments [5, 6]. Before adopting this approach, we need a better understanding of the relative importance of trans-diagnostic expressions of psychopathology and risk that precede the onset of the first full-threshold episode of a major mental disorder (which we will refer to as ‘caseness’).

Clinical staging models, such as employed in medicine, are increasingly utilized in psychiatry [2, 5, 6]. Essentially, a staging framework allows clinicians and researchers to determine where an individual is located on a continuum from stage 0 to stage 4. The earliest stages (0–2) for mental disorders are typically identified as evolving from as asymptomatic but at-risk state (e.g., family history of mental disorder), to having increasing numbers of non-specific symptoms or experiences, to presenting with attenuated (clinical high risk) or subthreshold syndromes, through to exhibiting a first full-threshold episode. Longitudinal monitoring of cohorts comprising youth with various ‘at risk’ presentations (the so-called ‘close-in’ strategy), indicates that 10–40% of individuals demonstrate subsequent onset of a mental disorder meeting diagnostic criteria over 12–36 months [7, 8]. Currently, it is unclear why only some individuals identified as being at risk of developing a mental disorder show transition to caseness and others who appear to be equally at risk do not [9]. Also, whilst family history of major mental disorders and/or the presence of subthreshold syndromes are associated with the development of full-threshold disorders, there is uncertainty regarding their specificity (e.g. a subthreshold depressive syndrome may be associated with subsequent onset of a range of full-threshold mood, psychotic or other diagnoses) [8]. Some investigators have suggested that inconsistent research findings regarding homotypic continuity (where family history, psychopathology or subthreshold conditions precede the onset of the corresponding full-threshold mental disorders) may indicate that certain phenomena associated with transition to caseness may operate trans-diagnostically. For example, sleep-wake cycle disturbances (possibly indicative of circadian dysrhythmias), attention and problem-solving difficulties (neurocognitive impairments) and symptoms such as distress, worry, feeling overwhelmed or rumination (so-called cognitive-emotional dysregulation) may be exacerbated prior to the onset of a wide range of mental disorders [8, 10].

Research in the field of early intervention in psychiatry has addressed different aspects of risk and clinical staging, but most publications report the evolution of specific disorders until recently [2, 5]. This is partly explained by the fact that many funding streams for mental health research and academic journals are geared towards promoting projects that focus on the evolution and outcomes of specific diagnostic categories. Furthermore, trans-diagnostic research has proven to be difficult to plan and undertake and the translation of findings to clinical settings has not always been apparent. As such, we suggest that for trans-diagnostic models to be adopted more often in research and clinical practice, it is necessary to clarify whether shared risk factors and/or symptom patterns examined in disorder-specific studies can be utilized to reliably determine trans-diagnostic caseness. For example, youth with a family history of a major mental disorder are known to be at increased risk of developing mental disorders than individuals without such a history [2, 5, 8, 9]. Also, Eaton [10] and others have independently reported that transition to a full-threshold disorder may be the consequence of the evolution of three early expressions of psychopathology that are linked, but not the same, namely: (a) the accumulation of non-specific symptoms (e.g. overall symptom burden); (b) the differential intensification of a subset of existing symptoms (e.g. as exemplified by a subthreshold syndrome); or (c) by the acquisition of new symptoms (e.g. a cluster of symptoms that may not be associated with a particular diagnostic class, but e.g. with a trait or dimension such as internalization). The latter possibility has not been studied as extensively as the other risk pathways in trans-diagnostic research, but recent advances in statistical modelling and data mining techniques are improving the possibility of identifying constellations of symptoms that might be associated with illness progression [2, 5, 8–10].

We decided to examine the likelihood of developing a mood or psychotic disorder in individuals in the peak age range for onset of major mental disorders according to their exposure to early expressions of psychopathology and risk (i.e. family history; total symptom load; subthreshold syndromes; and newly identified clusters of specific symptoms discovered via machine learning). The study draws on data from an established study of a community-residing cohort of twins and non-twin siblings that has included repeated cross-sectional mental health assessments throughout adolescence and early adulthood. Focusing on the assessments undertaken between the ages of about 15 and 25, we undertook a planned sequence of analyses (all of which were adjusted age, sex, twin status and, in some analyses, also for family history) to examine the following questions:

1. Does the likelihood of transition to caseness differ according to the presence or absence of early expressions of psychopathology and risk?
2. Is there a combination of early expressions of psychopathology and/or risk that offers an optimal classification of cases and non-cases?
3. Is there any benefit from using a machine learning approach, namely Chi-Square Automatic Interaction Detector (CHAID), in trans-diagnostic research compared with traditional approaches to determining caseness (i.e., positive and negative predictive values and accuracy of case classification)?

## Methods

### Overview of the Brisbane longitudinal twin study (BLTS)

Ethical approval was obtained for the Brisbane Longitudinal Twin Study (BLTS) from the Human Research Ethics Committee at the Queensland Berghofer Institute of Medical Research (references: EC00278 and P1212). The study follows Strengthening the Reporting of Observational Studies in Epidemiology guidelines (S1 Checklist). Additional descriptions of the protocol, procedures and data collection processes are provided in the (S1 File). Further details and research findings (including health service use and treatment profiles) are also available in other recent cohort publications [8, 11–13]. Here we provide only a precis of key information relevant to the current study.

Essentially, the BLTS is a community-based cohort study of twins and non-twin siblings, recruited via media appeals and word of mouth from 1992 onwards. Ethnically, the cohort reflects the population structure of the greater Brisbane area at the time of recruitment, with most participants of European ancestry and minorities of predominantly Asian ancestry [11, 12]. Individuals were eligible to join the cohort from age 12 onwards with written parental consent. However, the current article focuses only on data collated between the ages of about 15–25 years when participants completed a set of mental health and family history assessments. Repeated self-rating and interview assessments were performed during this peak age range for the onset of mood and psychotic disorders, and individuals who missed a follow-up could be invited to participate at the next wave [8, 11]. Due to the nesting of the data collection within a longitudinal framework, findings from recent cross-sectional assessments can be linked to those from earlier waves [11].

### Cohort eligible for this study

De-identified individual data were extracted from the BLTS dataset according to the following inclusion criteria:

- the cohort member had completed all self-ratings of psychopathology at a follow-up undertaken between age 15–19;
- the Composite International Diagnostic Interview (CIDI) [13] and family history of major mental disorders [14] assessments were available from the 19Up or 25Up follow-ups;
- age at onset of self-rated psychopathology, e.g. sub-threshold syndromes, and/or of the first full-threshold CIDI episode was recorded (or could be estimated from information available in the dataset).

Our aim was to explore symptoms and subthreshold syndromes that represented antecedents, not consequences, of any full-threshold syndromes identified. As such, individuals were excluded from the analyses if the age at completion of the CIDI assessment and/or estimated age at onset of any full-threshold syndrome *preceded* the age at completion of the symptom self-ratings. Likewise, we excluded individuals if insufficient data were recorded and/or ages at onset could not be estimated or specific age data were missing.

Using the above criteria, we identified that 1815 individuals (out of 2540 potential participants) met all the eligibility criteria. As shown in S1 Table, the excluded group hardly differed from the included cohort, except that the former was slightly older (mean age 26.9 versus 26.4 years; p<0.03).

### Assessments

1) Demography: key characteristics were recorded (see results).

2) Self-Rated Psychopathology: The box lists the 23 mood and psychotic symptoms/experiences included in three self-rating scales (Hypomanic-Like Experiences: HMLE; Psychotic-Like Experiences: PLE; Depressive-Like Experiences: DLE) [9]. The test-retest reliability of the self-ratings is good (inter-class correlations = 0.8) and the inter-rater reliability (weighted kappas) is about 0.75 for each subthreshold syndrome [15–19]. These self-report instruments were chosen as they are widely used to evaluate any psychopathology experienced by young people and the ratings can be examined from several perspectives (S1 File gives details of symptoms and rating scale properties, etc). First, the total number of items endorsed across all three rating scales can be used to estimate overall symptom load (Sx_Load). Second, item endorsements on a particular scale can be used to determine if an individual has experienced a pre-defined subset of symptoms that represent a sub-threshold syndrome (SubT) [8, 9]. Also, in this study, we used machine learning (see statistics section) to examine patterns of item endorsements across all three ratings to examine whether there is a cluster of symptoms that may represent a clinical phenotype for trans-diagnostic transition to caseness.

**Box. Description of self-rated symptoms.**

**Table:** 

Item Number	Item description	Item Number	Item description
	**Depressive Symptoms**		**Hypo/Manic Symptoms**
DLE1	Nervous/Tense	HMLE1	Feeling Elated
DLE2	Sadness/Depressed Mood	HMLE2	Increased Self-Esteem
DLE3	Feel Stressed	HMLE3	Need Less Sleep
DLE4	Feel Overwhelmed	HMLE4	Increased Psychomotor Speed (Speech)
DLE5	Loss of Confidence	HMLE5	Increased Activity (Physical)
DLE6	Hopelessness		**Psychotic Symptoms**
DLE7	Somatic Pain	PLE1	Thoughts Not Your Own
DLE8	Hypersomnia	PLE2	Third Party Auditory Hallucinations
DLE9	Fatigue	PLE3	Hearing Voices (when alone)
DLE10	Impaired Sleep (Quality)	PLE4	Feeling Threatened by Others
DLE11	Impaired Concentration	PLE5	Thinking People are Against You
DLE12	Anergia	PLE6	Thought Withdrawal

DLE: Depression-Like Experiences; HMLE: Hypomanic-Like Experiences; PLE: Psychotic-Like Experiences.

3) CIDI caseness: We applied established algorithms to CIDI assessment data to determine the presence or absence of a range of DSM-IV disorders and their age at onset [13]. For this study, we extracted data regarding the presence or absence of major depressive, hypo/manic, and/or psychotic syndromes that met CIDI criteria for caseness (see S1 File). Whilst we noted the co-occurrence of these syndromes, we did not explore diagnostic subgroupings separately (as this is a trans-diagnostic study).

4) Family History of Mood and Psychotic Disorders: Psychiatric histories in 1st and 2nd degree family members were identified using an online assessment based on the Family History Screen [14]. As with CIDI ratings, this study used the dichotomous ratings regarding the presence or absence of any family history (FH) of mood and/or psychotic disorders.

### Statistical analyses

Analyses were undertaken using RStudio (version 3.5.3) and SPSS (version 27.0). Additional details regarding statistical procedures are provided in the (S1 File); all reported analyses consider familial clustering [8, 11, 12].

Descriptive statistics are reported as means with standard deviations (M; SD), medians with inter-quartile ranges (IQR) and counts and percentages (for categorical variables). To explore the utility of FH, Sx_Load and SubT in differentiating between cases and non-cases, we used Bayes’ theorem to estimate the positive and negative predictive values (PPV; NPV) and their 95% confidence intervals (95% CI) [20]. Overall accuracy of each classification is reported as Area under the Curve (AUC) with 95% CI. The NPV, PPV and AUC were estimated for each variable separately (FH; Sx_Load; SubT) and then for combinations of variables (FH with Sx_Load; FH and SubT).

The above analyses used data about exposure to previously known antecedents of full-threshold disorders that are well described in the existing research literature (i.e., FH, Sx_Load, SubT). However, to examine whether a particular group of self-rated trans-diagnostic symptoms can discriminate cases from non-cases, we employed an analysis that generated a cluster of symptoms *de novo*. We used a machine learning approach to perform Chi-Square Automatic Interaction Detector (CHAID) analyses [21, 22]. We undertook two planned analyses of self-rated symptoms that allowed reporting of findings allow comparison with the previous analyses. The initial CHAID model included all 23 self-rated symptoms whilst the second model included FH as well as the self-rated symptoms. However, the latter analysis should be regarded as exploratory as preferably it should be undertaken in an entirely independent cohort.

Additional details about CHAID are provided in S1 File, but it is important to note that we applied recommended approaches for minimizing over-fitting of the models and for optimizing reliability and validity of the analysis. First, we created a single ‘variable of influence’ that accounted for individual differences in age, sex, and familial clustering (ie. age above or below the cohort median; male versus female; monozygotic versus other sibling status). This variable ensured that the CHAID model was adjusted for these characteristics (but avoided the need to over-ride the automatically determined maximum tree depth). Second, all CHAID analyses were cross-validated using train-test split evaluation with 50% split (i.e., the model was trained on a ~50% random sample of the cohort and then tested on the remaining half) and, in the results section, we report findings on the test set. We only report findings for the test models (as this is less vulnerable to over-fitting and offers a better estimate of validity and of performance of the machine learning algorithm). Third, we set a conservative level of statistical significance (Bonferroni adjusted p<0.01) for the inclusion of variables in the final tree structure. Fourth, the stopping criteria for tree generation were set *a priori* (S1 File details the rationale for the minimum node size for splitting or creation and notes maximum tree depth, etc.).

For readers less familiar with CHAID, we briefly summarize how to interpret the outputs. A CHAID analysis generates a bifurcating decision tree composed of a root node (the variable with the strongest association with the dependent variable and lowest p-value) which then branches and grows iteratively into internal and terminal nodes (the latter represent variables that carry maximum information). The order of importance of explanatory variables is represented by the tree structure and the percentages shown within the nodes are an indicator of the relevance of each characteristic as a primary predictor of CIDI status at follow-up (and PPV and NPV) [23]. In the current study, the PPV and NPV are reported for different combinations of specific symptoms identified in the classification trees. As the roots and nodes may link in different ways (following an ‘if-then’ type sequence), there may be more than one PPV or NPV associated with variables included in the tree. However, the reported AUC indicates the overall accuracy (performance) of the CHAID model. Tree building ends when p-values of all the observed independent variables are above the specified threshold for statistical significance, so the absence of any self-rated symptoms from the trees we report indicates that those items did not make a meaningful additional contribution to case classification. It should be noted that, as the tree diagrams for the ‘test set’ only include findings regarding root and terminal nodes with corrected p-values <0.01, we have not reported summary statistics for each root and node (this has been done to make the classification tree easier to interpret, but all these statistics are available upon request).

## Results

### Cohort characteristics

As shown in Table 1, the sample comprised 1815 participants (Female = 1050; 58%) with a mean age of 26.4 (S = 4.2); about 55% were single and 60% were in full-time employment.

**Table 1:** Summary of key characteristics of the study cohort (also see S1 and S2 Tables).

Characteristic	N = 1815
*DEMOGRAPHICS*^*a*^	
Mean Age in years (SD)	26.4 (4.2)
	***Number (%)***
Females	1050 (58%)
Educational Level: Secondary School only	327 (18%)
Full-Time Employment	1089 (60%)
Civil Status: Single	998 (55%)
Zygosity^b^	
Monozygotic Twins	509 (28%)
Dizygotic Twins	698 (38%)
Non-Twin Siblings	608 (33%)
*CLINICAL RATINGS*^*c*^	
Total Number of Self-Rated Symptoms (range 0–23)	
Mean (SD)	5.21 (4.35)
Risk Factors^c^:	***Number (%)***
High Symptom Load^d^	405 (22%)
Family History of Mental Disorder^e^	346 (19%)
Sub-Threshold Syndrome	383 (21%)
Family History & High Symptom Load	166 (9%)
Family History & Sub-Threshold Syndrome	198 (11%)
CIDI Diagnosis:	
Any Mood or Psychotic Disorder^f^	439 (24%)
> = 2 Disorders	129 (7%)

% reported to the nearest whole number; CIDI: Composite International Diagnostic Interview. SD: Standard Deviation.

^a^See S1 Table for comparison of included versus excluded cohort members.

^b^Odd numbers indicate only one co-twin from a twin pair was included.

^c^Categories are not mutually exclusive; Also, see S2 Table for details of inter-relationships between risk factors & CIDI diagnosis

^d^High Symptom Load defined as total number of self-rated symptoms greater than the cohort median score (= 5).

^e^Family History of major mood or psychotic disorder.

^f^Disorder refers to the presence of a depressive, hypo/manic &/or psychotic syndrome that met CIDI criteria for caseness.

On average, individuals self-reported the co-occurrence of 5.21 symptoms (SD = 4.35; median = 5); the distribution of scores for total number of self-rated symptoms is shown in S1 Fig. The prevalence of each self-rated DLE, HMLE, and PLE symptom ranged from 1–50% (see S2 Table). For all cohort members, the three most frequently endorsed items were hypersomnia (DLE8), feelings of elation (HMLE1) and impaired sleep quality (DLE10); the three least frequently endorsed items were third party auditory hallucinations (PLE2), thought withdrawal (PLE6) and hearing voices when alone (PLE3).

About 21% (n = 383) of the cohort had symptoms that met criteria for a > = 1 SubT and 346 (19%) individuals had a FH of a mood and/or psychotic disorder. Of these individuals, 198 (11%) reported both a SubT and FH. About 31% of the study cohort (n = 568) met criteria for > = 1 subsequent full-threshold CIDI diagnosis. The median age at onset for self-reported psychopathology, such as the first SubT, was about 16 years (IQR: 14–18) and the median age at first onset of a full-threshold syndrome meeting CIDI diagnostic criteria was about 20 years (IQR: 18–23).

### Early expressions of psychopathology and risk and subsequent CIDI caseness

Some data regarding the co-occurrence of FH, Sx_Load, and SubT with each other and with CIDI caseness are shown in Table 1, whilst PPV, NPV and AUC estimates are presented in Table 2 in the main text (additional raw data are provided in S3 Table in the supplementary materials). Regarding exposure to antecedents, it was additionally noted that 113 individuals identified as low risk for caseness (Low Sx_Load; SubT-; FH-) subsequently met criteria for a CIDI syndrome (6% of total cohort), whilst 78 individuals identified as high risk (High Sx_Load; SubT+; FH+) did not meet criteria for a CIDI syndrome during follow-up (4% of the total cohort) (see S3 Table).

**Table 2:** Classification of cohort members according to early expressions of psychopathology and risk and subsequent caseness: Positive and Negative Predictive Values (PPV; NPV), Area Under the Curve (AUC) and 95% Confidence Intervals (CI).

Risk Group		PPV	NPV	AUC
		(95% CI)	(95% CI)	(95% CI)
**Family History**		40.44	72.31	62.75
		(37.13, 43.85)	(70.83, 73.74)	(60.48, 64.98)
**Symptom Load**		44.60	81.61	63.14
		(42.55, 46.78)	(79.49, 83.56)	(60.87, 65.36)
**Subthreshold Syndrome**		55.19	77.23	**72.76**
		(52.43, 57.93)	(75.41, 78.95)	**(70.80, 74.65)**
**Symptom Load**	**FH-**	40.33	**83.33**	62.87
		(37.72, 42.95)	**(80.99, 85.44)**	(58.65, 66.94)
	**FH+**	52.87	76.52	63.26
		(49.35, 56.35)	(71.69, 80.75)	(60.54, 65.91)
**Subthreshold Syndrome**	**FH-**	44.91	79.73	69.24
		(41.04, 48.88)	(77.93, 81.42)	(66.62, 71.77)
	**FH+**	**60.10**	70.81	66.91
		**(54.55, 65.40)**	(67.48, 73.93)	(62.78, 70.86)

Estimates in bold represent the highest value for a parameter across all risk groups.

AUC: Area under the curve; FH: family history; NPV: Negative predictive value; PPV: Positive predictive value.

As shown in Table 2, case classification according to FH demonstrated a PPV of 40.44% (95% CI: 37.13, 43.85), a NPV of 72.31% (95% CI: 70.83, 73.74) and an AUC of 62.75% (95% CI: 60.48, 64.98). For case classification according to preceding Sx_Load, the PPV was 44.60% (95% CI: 42.55, 46.78), the NPV was 81.61% (95% CI: 79.49, 83.56) and the AUC was 63.14% (95% CI: 60.87, 65.36). For case classification according to preceding SubT, the PPV was 55.19% (95% CI: 52.43, 57.93), the NPV was 77.23 (95% CI: 75.41, 78.95) and the AUC was 72.76% (95% CI: 70.80, 74.65).

When case classification was explored for the different combinations of early expressions of psychopathology and risk factors, the NPV increased for Low Sx_Load and FH- (88.33%) and the PPV increased to 52.87 for FH+ and Sx_Load and to 60.10% for FH+ and SubT+. However, Table 2 demonstrates that all NPV and PPV estimates were in the moderate range and changes in the AUC for combined models were only marginal (with some improving but some declining).

### Classification tree models

Diagrams show trained classification trees run on the test set (hence sample size is about 50% of the study cohort) and comprises of roots and nodes that have statistically significant chi-square estimates and adjusted Bonferroni p values (these statistics are not shown here but are available from authors).

#### Test model 1: Self-rated symptoms

**Fig 1:**
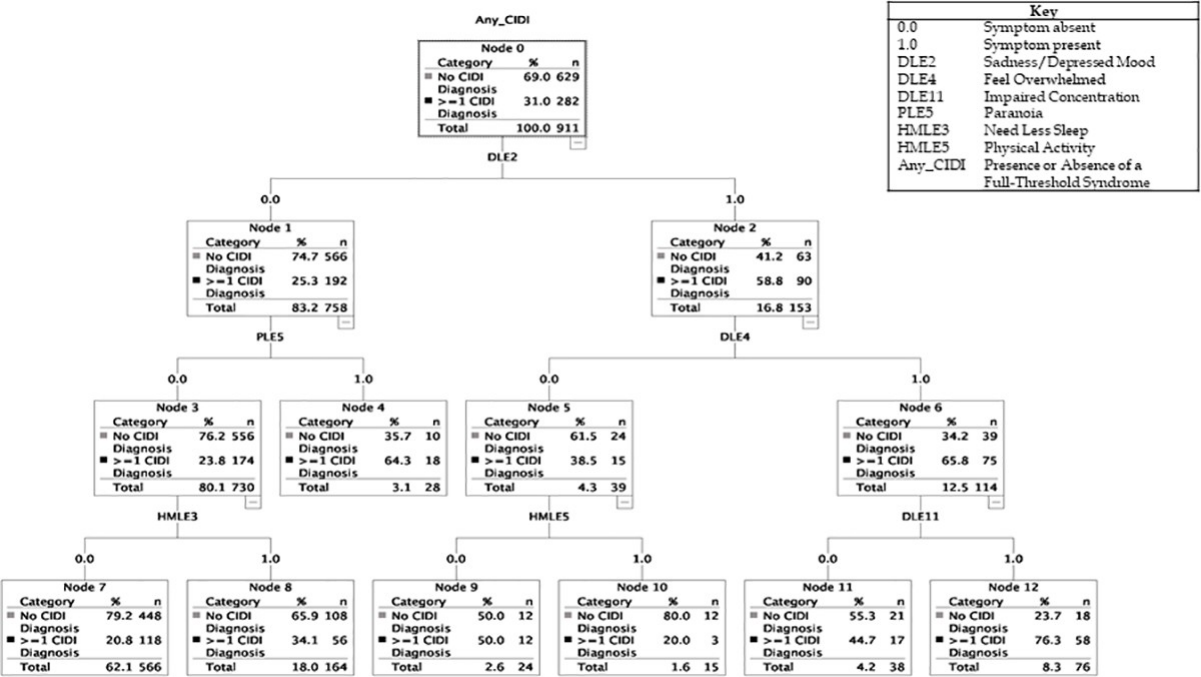
Classification tree for model 1: Self-rated symptoms, with adjustment for age, sex and familial clustering.

The classification tree identified that six self-rated experiences can be utilized to predict CIDI caseness (see Fig 1); the overall AUC for this model was 74.31% (95% CI: 72.72, 75.87).

The root node is represented by sadness/depressed mood (DLE2). The PPV for CIDI caseness was 76% (Node 12) in individuals who reported sadness (Node 2), feeling overwhelmed (DLE4; Node 6) and impaired concentration (DLE11; Node 12). In the absence of sadness (Node 1), CIDI caseness was more likely than not in those who reported paranoia (PLE5; Node 4). The NPV for not being a case was 79% (Node 7) and was best predicted by the absence of sadness (DLE2), paranoia (PLE5), or reduced need for sleep (HMLE3). Although physical activity (HMLE5) is included in the model, it appears to have limited utility for predicting caseness.

#### Test model 2: Self-rated symptoms combined with FH

**Fig 2:**
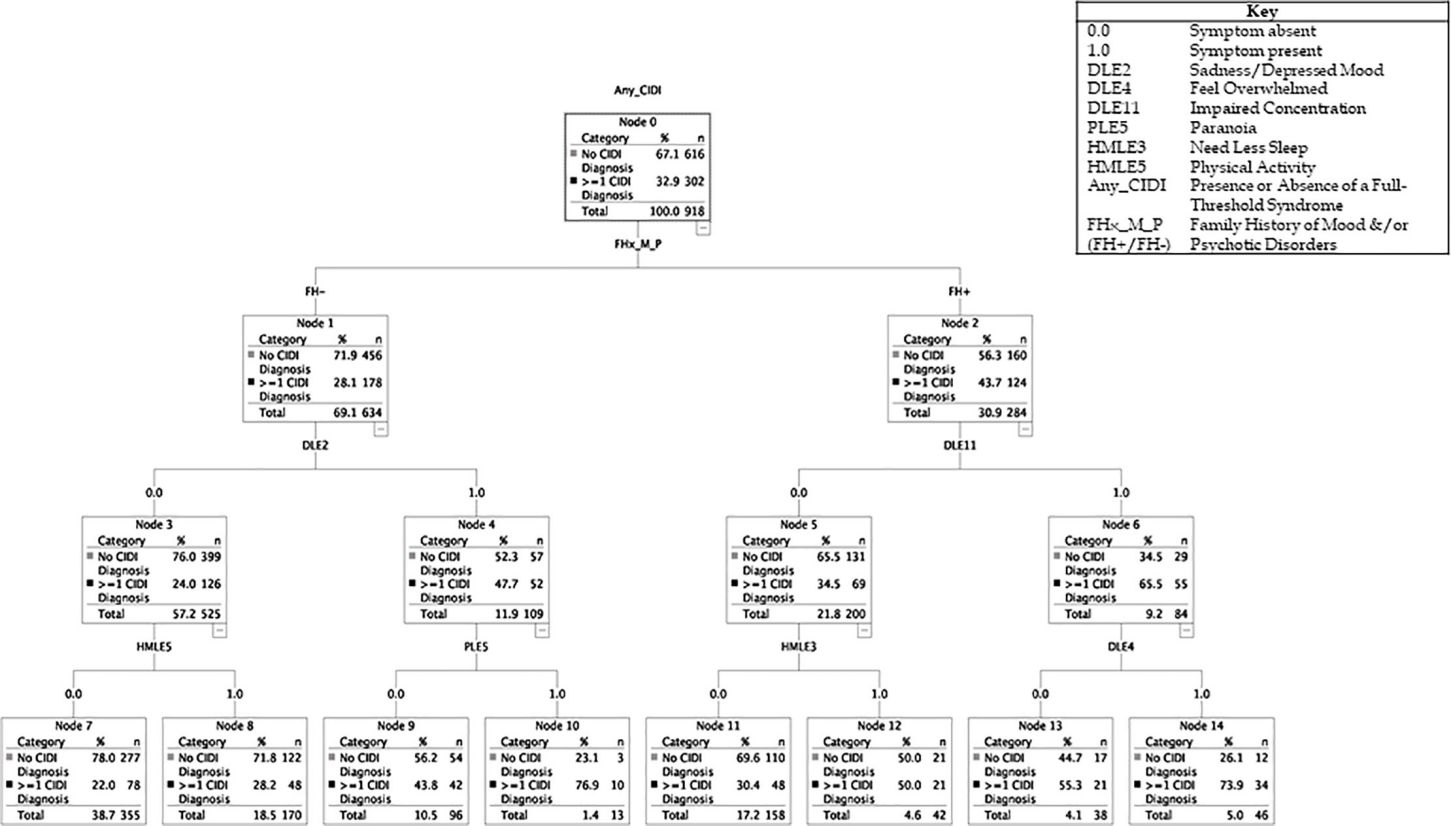
Classification tree for model 2: Family history combined with self-rated symptoms, with adjustment for age, sex and familial clustering.

It was interesting to note that the classification tree shown in Fig 2 indicates that the addition of FH into the CHAID analysis did not lead to a change in the self-rated symptoms included in the diagram; the overall AUC for this model was 75.23% (95% CI: 73.18, 77.21).

As shown in Fig 2, the PPV for CIDI caseness was 74% in FH+ individuals who self-reported impaired concentration (DLE 11; Node 6) and feeling overwhelmed (DLE4; Node 14). Further, the PPV for caseness was 77% in FH- individuals who self-reported sadness (DLE2; Node 4) and paranoia (PLE5; Node 10). The NPV was 78% in FH- individuals without self-reported sadness (Node 3) or increased physical activity (HMLE5; Node 7). In Model 2, HMLE5 (physical activity) and HMLE3 (need for sleep) appear to make limited contributions of to the test model.

## Discussion

This study of a community-residing cohort suggests that the self-reported presence of a FH of a major mental disorder, a SubT, and/or total symptom burden represent ‘pluripotent’ risk factors for subsequent clinical caseness during the peak age range for onset of mood and psychotic disorders. These findings confirm those of studies of similar community and clinical cohorts [5–10, 24–27]. Using PPV, NPV, and AUC as estimates for case identification and discrimination (Table 2), we noted that the characteristics that best predicted not being a case at follow-up were the absence of a FH of a major mental disorder alongside low levels of pre-existing psychopathology. Predictions of caseness (PPVs) were suboptimal for use in clinical settings and suggest that these early expressions of psychopathology and risk offer insufficient precision to be employed as trans-diagnostic screening tools without further revision. This view is confirmed by the AUC findings. Overall, we found that SubTs gave the highest accuracy for discriminating cases from non-cases (AUC 72.76%), however, this level of accuracy is below the 85% threshold which is typically regarded as acceptable for use in preventive medicine projects. Moreover, the AUC we reported is lower than reported in some other studies of SubTs (or related constructs such as attenuated syndromes) [28, 29]. However, those studies may be at risk of Berkson’s bias, as higher AUCs have only been noted by studies of clinical populations (e.g., transition to psychosis in specialist clinical settings) [28], rather than studies of trans-diagnostic transition in community settings [9]. A further issue was that the AUC for SubT did not improve when FH was considered (and the PPV decreased). These findings may initially seem counter-intuitive, but it should be born in mind that previous research suggests that the inter-relationships between these pluripotent risk factors are complex [28]. For example, in this and other studies, it has been shown that individuals with a FH are more likely to have an earlier age at onset of CIDI disorders than those without a FH [7]. Also, we noted that one in five of the sample experienced a SubT, whilst about one in ten experienced a SubT with a FH. So, as the accuracy of predictive models is affected by subgroup sample size (and our models were further adjusted for familial clustering), it is not surprising that there were small shifts in the apparent AUC, etc. Of course, this explanation needs testing in further research.

Given the above, the decision to explore if there is any added value from using a machine learning approach like CHAID is justified [30, 31]. We were especially interested in whether this approach might generate a constellation of symptoms not identified previously, and whether CHAID might help to establish the relative importance of individual and/or combinations of self-rated symptoms that predict future CIDI caseness. The classification trees reported (Figs 1 and 2) provide novel insights into symptom constellations and their associations with FH that we were unlikely to uncover using disorder-specific ratings of symptoms or risk syndromes. The most notable finding was that the CHAID analysis identified that four transdiagnostic symptoms, namely sadness, feeling overwhelmed, impaired concentration and paranoia, may improve the prediction of caseness (compared with other early expressions of psychopathology). Also, the AUC for the classification tree was marginally improved when FH was included (which was not typically the case in the estimates in Table 2). It can be argued that the overall accuracy of the two CHAID models (median AUC ~74.5%) showed only an incremental improvement over more traditional statistical approaches to established markers of risk and psychopathology (median AUC ~64%). However, the CHAID models were associated with improvements in PPV (compared to those in Table 2) and the machine learning algorithm generated different PPV or NPV estimates for different configurations of symptoms. For example, the PPV for CIDI caseness was 76% in individuals who reported sadness, feeling overwhelmed and impaired concentration, whilst the PPV was 77% in individuals who self-reported sadness and paranoia. The CHAID analyses also highlighted that some symptoms that are integral to the models (need for sleep and physical activity) may be more useful predictors of CIDI classification if they are *not* endorsed (as evidenced by the NPV for not being a case) rather than if they *are* endorsed.

Notably, none of the four most important symptoms identified in the classification tree is uniquely associated with a diagnosis of any mood or psychotic syndrome [28, 32–34]. However, these experiences do represent markers of neurocognitive impairment (concentration) and cognitive emotional regulation; also, two symptoms, namely physical activity and need for sleep, may be associated with circadian rhythms. The association between this cluster of symptoms with future caseness will not surprise clinicians, as several studies demonstrate that transition to psychotic disorders is often associated with the co-occurrence of mood and anxiety symptoms and/or full-threshold depression (or vice versa) [28, 29]. Observers might argue that most of the symptoms identified via CHAID are linked particularly to mood disorders, but this would be a misrepresentation of the findings as the annotations of DLE, HMLE and PLE were instituted by researchers (primarily to delineate different subthreshold syndromes) and clinicians (as screening tools for putative diagnoses), not by participants completing the self-ratings [7, 8, 15–19]. So, it must be borne in mind that participants had no prior knowledge of how the researchers subcategorize the ratings or that this study would examine three different early expressions of psychopathology. As such, we do not think there was any self-serving bias in the pattern of self-reported experiences. Regarding the relative importance of these symptoms, it is important to note that their ability to discriminate cases from non-cases and predict onset of a future CIDI syndrome was not associated with their prevalence. The four key symptoms in the classification tree are not the most frequently reported self-rated items in this cohort (S3 Table). So, it may be that the co-associations between trans-diagnostic self-ratings (here, ratings of sadness, feeling overwhelmed, impaired concentration and paranoia, with or without their links to family history) could represent overarching or ‘meta’ phenomena associated with future CIDI caseness [32–34].

### Limitations

We acknowledge that the findings must be replicated in independent samples and using other assessment tools and/or analyses. For example, although there is evidence to support the generalizability of our findings (i.e., prevalence rates for risk factors and CIDI diagnoses were similar to other studies) and we adjusted analyses for age, sex and familial clustering, it would be unsafe to assume this community cohort (a third of which comprised monozygotic twins) is representative of other cohorts [7, 9]. Of more concern is the possibility that CHAID analyses may ‘overfit’ the classification trees. Even though we took precautions to minimize this risk and only report the cross-validated models, we acknowledge that the findings of the second CHAID analysis (which included FH) must be treated with caution as the analysis and findings require replication in an independent sample. We also relied on self-ratings to assess expressions of psychopathology and risk. Self-report of FH can be unreliable, and unlike other similar recent studies, we did not incorporate polygenic risk scores (PRS) [9]. Also, we relied on simple categorical self-ratings of symptoms/experiences, rather than more sensitive, dimensional, or temporal evaluations. Lastly, the BLTS uses repeated cross-sectional assessments, which could be used to make more subtle examinations of longitudinal course of phenomenology using survival or temporal network analyses and other dynamic explorations of risk [30, 31].

### Implications & comments

Finally, we consider the findings in the broader context of preventive psychiatry. Prevention strategies are informed by observational research on risks and adverse outcomes [2, 5, 28, 29, 35–39]. However, as highlighted by Cuijpers [25], we lack a detailed understanding of the exact pathways leading to mental disorders. Further, one risk factor alone is rarely sufficient to produce or predict disorder and most known risk factors have low specificity [2, 7–9, 25, 27–29]. To enhance options for prevention, we need greater knowledge not only of which ‘at risk’ individuals are most likely to transition to full threshold caseness, but also to develop screening procedures that capture the warning signs and symptoms during the specific timeframe when a ‘risk syndrome’ is most likely to evolve into a severe, impairing, or distressing condition [29–31]. The need to extend trans-diagnostic approaches is recognized in the literature [2, 5, 27, 39] and in published protocols for future prospective research [16, 35, 36, 38]. These all acknowledge that FH is an established marker of risk and emphasize that there is no rationale to recommend discontinuing its recording. However, they are less clear on other elements to include in screening. To date, early expressions of psychopathology or of clinical stage have either employed assessments of selected ‘clinical high risk’ syndromes [29] or, less frequently, micro-level assessments of specific symptoms as part of a disorder-specific project [37]. Unsurprisingly, low levels of symptoms have a high NPV, but the PPV of symptom load in this and other studies does not indicate that accumulation of symptoms can be translated into a risk assessment tool. SubTs have utility, but the definitions of different ‘risk syndromes’ are highly inconsistent and lack specificity; also, the increasing wish to undertake screening in community rather than clinical populations may limit their value. For example, a recent community study demonstrated that, whilst clinical high-risk states for psychosis have predictive validity for the onset of full-threshold psychotic disorders, the subthreshold antecedent conditions were relatively rare in the general population [40]. As such, the investigators suggested that a comprehensive prevention strategy with a focus on broader psychopathology may be more effective than a disorder-specific approach for achieving population-based improvements in prevention of major mental disorders [40]. Our study represents one of the first empirical reports to suggest novel, but discrete subsets of symptoms may have some utility in such a broad screening approach. We stress that the overall accuracy of the models reported here does not mean we regard the currently identified symptoms represent the optimal combination. However, a model comprising four trans-diagnostic symptoms appears to offer equivalent or better prediction of caseness than other established measures.

## Conclusions

Translation of findings into meaningful preventative or clinical interventions is never a given in staging and risk research [21, 22, 25, 40]. However, decision tree algorithms can help to evaluate pluripotent risk factors and accomplish the goals of trans-diagnostic research more effectively because the approach can have greater accuracy and makes fewer assumptions than most multivariate models [39]. Critically, many previous publications presume there are linear relationships between dependent and independent variables and fail to take account of the contingent (“if-then”) nature of clinical risk and its assessment [34]. A potential reason for considering use of CHAID in the future is that the approach might allow identification of homogeneous subgroups from within heterogeneous trans-diagnostic populations. The advantage of this strategy is that it is ‘diagnostically agnostic’ and is more likely to identify symptom constellations that have not been identified previously. This is important for genetic or biological research where investigators are keen to find new ways of dissecting diagnostic or clinical stage entities into homogeneous ‘correlated phenotypes’ or ways of supplementing the clinical stage/diagnosis categories with quantitative measures of phenotypic traits [8, 38, 41, 42]. The current study offers a template for future studies, which may incorporate symptom ratings alongside PRS or putative phenotypic markers (such as actigraphy-based rest-activity rhythms) to generate new screening instruments and risk prediction algorithms. Furthermore, whilst we focus mainly on selected intrinsic markers of vulnerability, we believe further research can incorporate other intrinsic dimensions (such as personality traits, functioning) and extrinsic markers, such as life events and adversity into a more comprehensive model [43].

In summary, we suggest that classification tree analysis of pluripotent risk appears to offer a useful tool for youth mental health research as it could potentially inform the development of more sophisticated screening procedures, selection of more nuanced or multi-model trans-diagnostic preventative interventions and promote an integrated science research on high-risk phenotypes within populations with early expressions of psychopathology.

## Supporting information

S1 Checklist Checklist of items that should be included in reports of cohort studies. (DOC)

S1 File Additional methodological details. (DOC)

S1 Fig Proportion of study cohort vs. total symptom score (range 0–23). (DOC)

S1 Table Comparison of cases included in the study cohort (n = 1815) with excluded cases (n = 725). (DOC)

S2 Table Prevalence of individual items. (DOC)

S3 Table CIDI diagnoses, sub-threshold syndromes, family history and symptom load for the study cohort. (DOC)
