# Supplementary material for: Early expressions of psychopathology and risk associated with trans-diagnostic transition to mood and psychotic disorders in adolescents and young adults
Source: PLoS One. 2021 Jun 4;16(6):e0252550. doi: 10.1371/journal.pone.0252550 (PMC8177455; doi:10.1371/journal.pone.0252550)
Supplement: S1 File — (DOC) [file pone.0252550.s002.doc]

**S1 File. Additional methodological details**

*Background Details of Brisbane Longitudinal Twin Study (BLTS)*

Detailed descriptions of the BLTS protocol and procedures (e.g. data encryption and management of anonymized survey information), recruitment strategies, and all the assessments undertaken in different waves are available elsewhere (e.g. Wright & Martin, 2004; Couvy-Duchesne et al, 2018; Mitchell et al, 2019; Scott et al, 2020). To summarize, this is a community-based cohort study of twins and their non-twin siblings living in the greater Brisbane area who were recruited via media appeals and word of mouth. Participants were included from the age of 12 onwards and recruitment was undertaken continuously between 1992 and 2015, with repeated follow-up waves every three or so years. Ethnically, the cohort reflects the population structure of Queensland at the time of recruitment, with most participants of European ancestry and minorities of predominantly Asian ancestry.

Written informed consent was obtained from potential participants (if aged >=18) or a parent (if aged <18 years). Individuals were excluded if parental report indicated a history of head injuries, neurological or pre-existing psychiatric conditions, substance misuse, and/or taking medications with significant central nervous system effects. An individual twin could be included even if their co-twin was ineligible or declined study participation. Follow-up assessments were coordinated to ensure follow-up waves began with interviews of older cohort members and moved towards younger members. Individuals who missed one follow-up cold be interviewed at the next wave.

As shown in the Figure, more recent follow-ups (waves) have increased the number of ratings of mental health and psychological well-being, etc. The follow-up assessments relevant to this article were undertaken from 2009 onwards (for protocol details see: Couvy-Duchesne et al, 2018; Mitchell et al, 2019). These waves, referred to as 19Up and 25Up, have included mental health assessments such self-report questionnaires about psychological distress and help-seeking and most recently, an assessment of full-threshold mental disorders meeting recognized diagnostic criteria and of family history of mental disorders.

Study Sample

The present study received ethical approval from the Human Research Ethics Committee at the Queensland Institute of Medical Research (QIMR) and is co-ordinated by QIMR Berghofer Institute in Brisbane in conjunction with the Brain and Mind Centre (BMC) at The University of Sydney. De-identified individual data used were extracted from the BLTS dataset according to the following eligibility criteria: the individual had completed an assessment of the self-report rating scales of mental health symptoms (see below) since 2009 and that data regarding CIDI and family history of mental disorders were available from the 19Up or 25Up follow-up respectively. If no CIDI assessment was completed, the individual was ineligible for this study. Also, individuals were excluded from the current project if they had not participated in all the core study assessments and/or the only available symptom self-ratings were recorded after the CIDI assessment (or receipt of a CIDI diagnosis) and/or the timing or sequence of completion of ratings was unclear. Individuals with sporadic missing data (e.g. 1-2 item ratings from the self-report scales or family history assessment) were eligible for inclusion; in these circumstances, missing data were assumed to indicate the absence or negative endorsement of an item (we identified only 13 individuals with missing items scores).

Assessments

1) Demographics: we recorded information regarding age (at completion of CIDI assessment), sex, zygosity, educational status and living situation.

2) Self-Report Ratings of Symptoms and Sub-Threshold Syndromes

We extracted data on self-ratings of symptoms and of the associated subthreshold syndromes and noted the age at onset of the latter (or the age of recording of the self-ratings if no subthreshold syndrome was recorded). The total number if symptoms was recorded for all self-rating scales and then the presence or absence of any subthreshold syndrome(s) was estimated using scores from each singular scale. The criteria used for subthreshold syndrome closely resemble those used for identifying individuals who meet ‘at risk’ criteria for mood or psychotic syndromes (e.g. Bechdolf et al, 2014; Lewinsohn et al, 2004; Yung et al, 2009). We have established that the test-retest reliability of the ratings is good (inter-class correlations= 0.8) (Scott et al, 2017) and when ratings of the same items are completed by a researcher the inter-rater reliability (weighted kappas) is about 0.75 for each subthreshold syndrome (Carpenter et al, 2019). The assessments include ratings of the following:

1. Hypomanic-Like Experiences (HMLE) were assessed by five symptoms included in the ‘BMC Hypomania/Activation Assessment’. The items are partly derived from symptoms included in in ‘Bipolar at Risk’ (BAR) criteria (originally described by Bechdolf et al (2014) and updated by Scott et al (2017) and that feature in the Altman Self-Rating Mania Scale (Altman et al, 1997). The HMLE items mirror DSM-IV criteria (e.g. elated mood, increased psychomotor activity, reduced need for sleep, etc.) and the tool is used to facilitate assessments of youth with hypomanic symptoms, bipolar spectrum or other presentations such as brief hypomanic episodes meeting the Zurich criteria (Angst et al, 2003; ibid, 2010). The criteria used for subthreshold syndrome was >2 days for the co-occurrence of all five symptoms listed.
2. Psychotic-Like Experiences (PLE) were assessed using the ‘BMC Psychosis Assessment’ that comprises of six items that assess the three sub-types of positive psychotic-like experiences most strongly associated with distress and poor functioning: bizarre experiences, perceptual abnormalities, and persecutory ideas (Yung et al, 2003). Item wordings represent minor modifications of questions that identified individuals at high risk of developing a psychosis in a community-based study of 875 school students aged 15-16 years (Yung et al, 2009). The criteria used for subthreshold syndrome was >2 days for the co-occurrence of at least two of the symptoms listed (which is similar to the criteria used for ‘BLIPS’: brief time-limited intermittent psychotic symptoms (Winton-Brown et al, 2011)).
3. Depressive-Like Experiences (DLE) were assessed using the 12-item version of the Somatic and Psychological Health Report (Hickie et al. 2001), which assesses the occurrence over the past few weeks of a range of somatic (e.g. Prolonged tiredness after activity; Waking up tired) and psychological symptoms (e.g. Feeling unhappy and depressed; Everything getting on top of you). The SPHERE was preferred to other putative measures of DLE as it may improve the detection of subthreshold syndromes in young males (compared to other similar tools) and has previously been used to explore subthreshold phenotypes of depression (Scott et al, 2018). The criteria used for subthreshold syndrome was the simultaneous presence of a ‘PSYCH-6’ sub-scale score>=2 and a ‘SOMA-6’sub-scale score>=3 for two or more weeks. These criteria are similar to those reported by several research groups (e.g. Lewinsohn et al, 2004).
4. Screening for Family History of Mental Disorders

The psychiatric health history of 1^st^ and 2^nd^ degree family members was assessed using an online assessment based on the version of the Family History Screen reported by Milne et al (2008; 2009). In the current study we used only the ‘dichotomous’ scores, which define probands as ‘family history positive’ if one or more of a proband’s first-degree family members had a positive history of a major mood or psychotic disorder (namely, depression, hypo/mania, bipolar, psychotic and/or schizophrenic disorders) and has received treatment for this condition.

1. Composite International Diagnostic Interview

The CIDI is widely used to determine ‘caseness’ and age at onset for a range of DSM-IV disorders (Kessler et al, 2004). The CIDI was undertaken by interview in the earliest follow-ups of the BLTS, but more recently via an online assessment programme. There are relatively few skip questions, but diagnoses can only be confirmed after further input from researchers who apply a scoring algorithm to the data to allow DSM IV diagnoses to be derived (Couvy-Duchesne et al, 2018). In the current study we focus on mood and psychotic disorders, so we extracted data regarding diagnoses of depressive, manic and hypomanic episodes according to core diagnostic criteria (A and B items of the DSM IV), but without enforcing exclusions (that no longer exist in DSM-5) e.g. bereavement in depression (Scott et al, 2018; ibid, 2020). It should be noted that the CIDI assessment we employed does not evaluate all the psychotic symptoms described in diagnostic criteria for the DSM (e.g. ratings are lacking for negative symptoms). As such, a full threshold psychotic syndrome was defined as present if the individual manifested severe and persistent core positive symptoms (delusions and/or hallucinations).

| **Figure: Overview of BLTS assessment waves (adapted from Couvy-Duchesne et al, 2018 and Mitchell et al, 2020):** the boxes next to each follow-up point identify the key themes and topics covered (with selected examples of ratings used reported in parentheses). The study includes cross-sectional & longitudinal assessments.  Note: Sample size in only indicative and does not consider ratings that were incomplete or invalid and/or that some waves are continuing to recruit new participants. | | | |
| --- | --- | --- | --- |
| 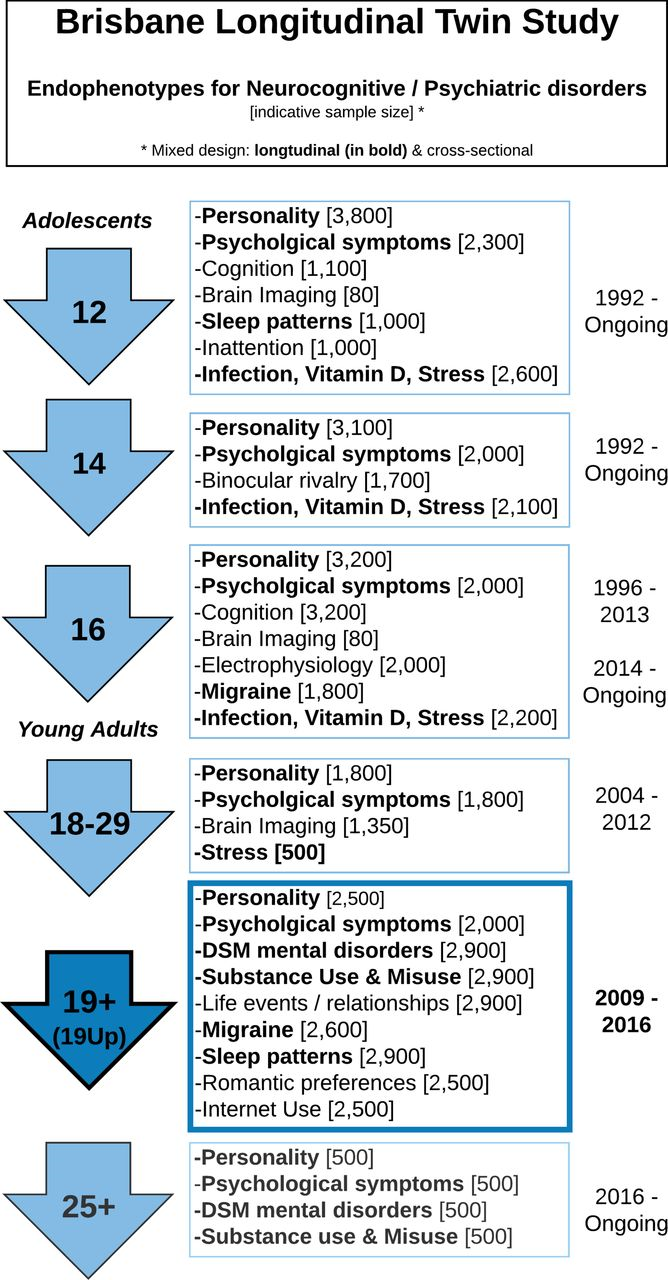 | 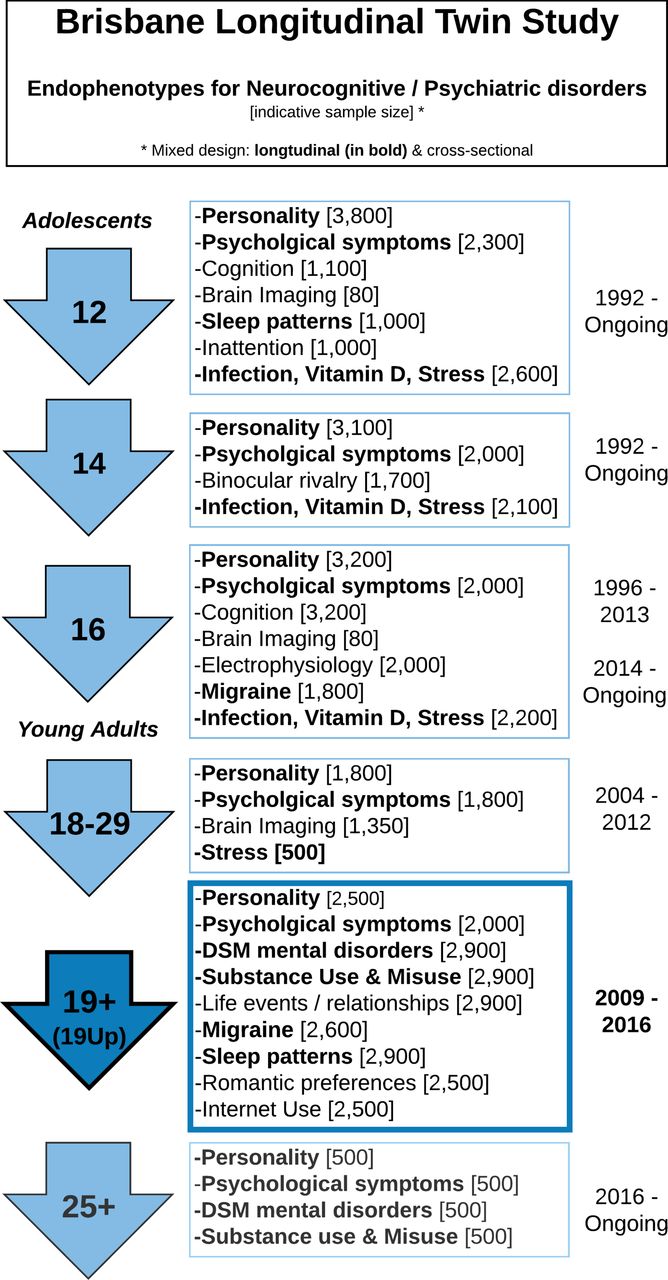 |  | |
|  | Personality [2500]  **Psychological Symptoms** [2000]  **CIDI- Mood & Psychosis** [~1900]  Other ratings [>2000] e.g. ASUD  Life events/relationships  Migraine  Sleep patterns  Internet Use  2009 -2016 |  | |
|  | Personality [2500]  **Psychological Symptoms** [2000+]  **Family History of Mental Disorders** [~1900]  Other ratings [>2000] e.g.  Inattention study  Melanoma  2016 -2020 |  | |
| Longitudinal, assessments have included: Mental health (self-ratings of hypo/manic, psychotic and depressive symptoms e.g. SPHERE); Personality traits e.g. neuroticism-extraversion-openness inventory (NEO), Junior Eysenck personality questionnaire (JEPQ); vitamin D; infections (antibodies); etc.  Cross-sectional assessments have included: Composite International Diagnostic Interview (at 19Up); cognition (verbal, performance IQ, working memory, information processing), binocular rivalry (rivalry rate) & brain imaging (multimodal MRI) at 12, 14 & 16 respectively; substance use (alcohol, tobacco, recreational drugs); hair cortisol; sleep patterns (actigraphy);life events/social support/ relationships (e.g., early home environment, family relationships, traumatic events, socioeconomic factors). | | |  |

Statistical Analyses

In this supplementary material, we comment only on risk rates (prevalence ratios adjusted for age, sex and zygosity) and CHAID analyses.

1. Positive and negative predictive values & Accuracy of classification of cases and non-cases-

The positive and negative predictive values (PPV; NPV) are the proportions of positive and negative results in [statistics](https://en.wikipedia.org/wiki/Predictive_value_of_tests) and [diagnostic tests](https://en.wikipedia.org/wiki/Diagnostic_test) that are [true positive](https://en.wikipedia.org/wiki/True_positive) and [true negative](https://en.wikipedia.org/wiki/True_negative) results, respectively. The PPV and NPV describe the performance of a diagnostic test or other statistical measure such as classification. A high result can be interpreted as indicating the accuracy of such a statistic. Unlike the [true positive rate](https://en.wikipedia.org/wiki/True_positive_rate) and [true negative rate](https://en.wikipedia.org/wiki/True_negative_rate), the PPV and NPV are not intrinsic to the test, but they depend also on the [prevalence](https://en.wikipedia.org/wiki/Prevalence). Both PPV and NPV can be derived using [Bayes' theorem](https://en.wikipedia.org/wiki/Bayes'_theorem) (Lee, 2012):

**P(A ∣ B) = P(B ∣ A)P(A) / P(B)**

where A and B are two events and P(B) ≠ 0

P(A ∣ B) is the [conditional probability](https://www.thoughtco.com/compute-probability-of-intersection-3126565) of event A occurring given that B is true.

P(B ∣ A) is the conditional probability of event B occurring given that A is true.

P(A) and P(B) are the probabilities of A and B occurring independently of one another (the marginal probability).

In the current study, this can be represented as:

*Positive predictive value*: probability of a CIDI syndrome in the presence of a risk factor and/or psychopathology.


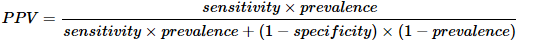


*Negative predictive value*: probability that a CIDI syndrome is not reported in the absence of a risk factor and/or psychopathology.


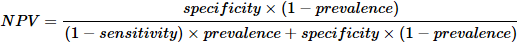


*Accuracy*: overall probability that a patient is correctly classified.

= Sensitivity × Prevalence + Specificity × (1 − Prevalence)

In this study: 95% confidence intervals for the predictive values are the standard logit confidence intervals (Mercaldo et al. 2007); whilst 95% confidence intervals for accuracy are "exact" Clopper-Pearson confidence intervals (Altman et al, 2000).

1. Classification Tree -

Data mining techniques have increasingly been used for finding and describing structural patterns in health and illness-related databases. A decision tree is a frequently used data mining method developed from machine learning theory that can generate classification, prediction, and regression models from data that are non-linear and/or derived from different measurement scales (Kass, 1980). Popular approaches, such as Chi-Square Automatic Interaction Detector (CHAID), represent non-parametric multivariate analyses that can identify robust data patterns that often remain undetected by traditional logistic regression or discriminant function analyses (Steadman et al, 2000). Conceptually, the decision tree is a sequential model consisting of a set of if-then rules for the partition of heterogenous input data into groups which are homogenous regarding the dependent/outcome variable categories (i.e. presence or absence of >=1 CIDI diagnosis in this study).

Decision trees based on CHAID use a systematic algorithm to detect associations between a categorical dependent variable and multiple independent nominal or ordinal variables. The ‘supervised learning’ algorithm minimises variations of the dependent variable within groups and maximises it between groups which enables detection of the strongest possible associations between predictors and the dependent/outcome category (Biggs et al, 1991). The relevant independent variables are selected hierarchically from the input dataset such that the first independent variable included in the tree (the root node) has the strongest association with the dependent variable and the highest level of statistical significance (i.e. the lowest p-value). The order of importance of explanatory variables is explicitly represented by the tree structure, and tree building ends when p-values of all the observed independent variables are above the specified threshold for statistical significance (usually an alpha level of 0.05, corrected for the number of statistical tests within each predictor using a Bonferroni multiplier that adjusts all p-values for multiple testing (Kass, 1980)). The stopping criteria for tree generation are selected *a priori* with minimum node size for splitting (parent node) usually being about twice the minimum leaf size for creation of a node (child node). Also, the analytic programme sets automatic criteria for maximum depth for the tree structure (which is 3 for CHAID models).

We were mindful that the overall prevalence of individual psychotic symptoms across the cohort was much lower than other symptom subtypes, so we set the parent and child node minimum sizes at 50 and 25 respectively and set the maximum tree depth at four. This was done to (a) increase the possibility of including any psychotic symptoms in the classification tree and (b) enhance the prospects of uncovering any influence of key demographic or risk variables. However, to balance these decisions and considering that we planned and tested two tree models on this cohort, we set a more conservative level for statistical significance, namely, the Bonferonni adjusted p-value at <0.01 (although we highlight a small number of findings of borderline statistical significance).

Raw data (see supplementary Table 2S) gives base rates/subgroup sizes for risk and outcome variables in the extracted dataset. On inspection, we determined that it was unrealistic to try to examine multiple exposures, such as multiple family history (FH in >1 first degree relative), presence of >1 subthreshold syndrome and/or multimorbidity of full-threshold syndromes (i.e. presence of >1 CIDI diagnosis).

We built the classification trees using split-half cross-validation (which is the recommended procedure as this allows an optimal tree model to be constructed in a training set and then verified in a test set). In this approach, the tree is built on the training subset and the performance of that tree is assessed on the testing subset. As per standard approaches, we focus our reporting and discussions on the ‘test’ trees which offer graphical representations of a series of decision rules. These classification trees are composed of a root node which branches and grows iteratively into a set of internal nodes, and a set of terminal nodes which represent the variables that carry maximum information. All other remaining variables are rejected from the model. Internal nodes represent groups that can be further split (into child nodes) to improve homogeneity, whilst terminal nodes comprise a group that is as homogeneous as is possible (with respect to the dependent variable), i.e. the model will not benefit from these terminal nodes being split further. The importance of the variable in a terminal node is reported as a percentage and this indicates the relevance of that characteristic as a primary predictor of the outcome (and it is argued that these represent positive or negative predictive values).

References

Altman DG, Machin D, Bryant TN, Gardner MJ (Eds) (2000) Statistics with confidence. 2^nd^ Edition. London: BMJ Books.

Altman EG, Hedeker D, Peterson JL. The Altman Self-Rating Mania Scale. Biol Psychiatry 1997; 42:948–55.

Angst J, Gamma A, Benazzi F, Ajdacic V, Eich D, Rössler W. Toward a re-definition of subthreshold bipolarity: epidemiology and proposed criteria for bipolar-II, minor bipolar disorders and hypomania. J Affect Disord. 2003 Jan;73(1-2):133-46.

Angst J, Cui L, Swendsen J, Rothen S, Cravchik A, Kessler RC, Merikangas KR. Major depressive disorder with subthreshold bipolarity in the National Comorbidity Survey Replication. Am J Psychiatry. 2010 Oct;167(10):1194-201.

Bechdolf A, Ratheesh A, Cotton SM, Nelson B, Chanen AM, Betts J, et al. The predictive validity of bipolar at-risk (prodromal) criteria in help-seeking adolescents and young adults: a prospective study. Bipolar Disord. 2014 Aug;16(5):493-504.

Biggs D, De Ville B, Suen E. (1991) A method of choosing multi-way partitions for classiﬁcation and decision trees. J. Appl. Stat. 18, 49–62.

Carpenter JS, Iorfino F, Cross S, Nichles A, Zmicerevska N, Crouse JJ, Palmer JR, Whitton AE, White D, Naismith SL, Guastella AJ, Hermens DF, Scott J, Scott EM, Hickie IB. Cohort profile: the Brain and Mind Centre *Optymise* cohort: tracking multidimensional outcomes in young people presenting for mental healthcare. BMJ Open. 2020 Mar 29;10(3):e030985

Couvy-Duchesne B, O'Callaghan V, Parker R, Mills N, Kirk KM, Scott J, et al. Nineteen and Up study (19Up): understanding pathways to mental health disorders in young Australian twins. BMJ Open. 2018 Mar 17; 8(3):e018959.

Greenland S. Interpretation and choice of effect measures in epidemiologic analyses. Am J Epidemiol. 1987 May;125(5):761-8.

Hickie IB, Davenport TA, Hadzi-Pavlovic D. Development of a simple screening tool for common mental disorders in general practice. Med J Aust 2001;175(Suppl): S10–17.

Iorfino F, Scott EM, Carpenter JS, Cross SP, Hermens DF, Killedar M, et al. Clinical Stage Transitions in Persons Aged 12 to 25 Years Presenting to Early Intervention Mental Health Services with Anxiety, Mood, and Psychotic Disorders. JAMA Psychiatry. 2019 Aug 28. 76(11):1167-75.

Kass G. (1980) An exploratory technique for investigating large quantities of categorical data. Appl. Stat. 29, 119–127.

Kessler RC, Abelson J, Demler O, Escobar JI, Gibbon M, Guyer ME, et al. Clinical calibration of DSM-IV diagnoses in the World Mental Health (WMH) version of the World Health Organization (WHO) Composite International Diagnostic Interview (WMHCIDI) Int J Methods Psychiatr Res. 2004;13(2):122–139.

Lee, Peter M (2012), "Bayesian Statistics: An Introduction," 4th edition. London: John Wiley.

Lewinsohn PM, Shankman SA, Gau JM, Klein DN. The prevalence and co-morbidity of subthreshold psychiatric conditions. Psychol Med. 2004 May;34(4):613-22.

Mercaldo N, Lau K, Zhou X (2007) Confidence intervals for predictive values with an emphasis to case-control studies. Statistics in Medicine 26:2170-2183.

Milne BJ, Moffitt TE, Crump R, Poulton R, Rutter M, Sears M et al. How should we construct psychiatric family history scores? A comparison of alternative approaches from the Dunedin Family Health History Study. Psychol Med. 2008; 38(12):1793-1802.

Milne B, Caspi A, Crump R, Poulton R, Rutter M, Sears M, Moffitt T. The validity of the family history screen for assessing family history of mental disorders. Am J Med Genet. 2009 Jan 5;150(1):41-9.

Mitchell B, Campos A, Renteria M, Parker R, Sullivan L, McAloney K, et al. Twenty-Five and Up (25Up) Study: A New Wave of the Brisbane Longitudinal Twin Study. Twin Res Hum Genet. 2019 Jun; 22(3):154-163.

Scott J, Martin N, Parker R, Couvy-Duchesne B, Medland S, Hickie I. Prevalence of self-reported subthreshold phenotypes of major mental disorders and their association with functional impairment, treatment and full-threshold syndromes in a community-residing cohort of young adults. Early Interv Psychiatry. 2020. Feb 12. [Epub ahead of print].

Scott J, Davenport TA, Parker R, Hermens DF, Lind PA, Medland SE, Hickie IB. Pathways to depression by age 16 years: Examining trajectories for self-reported psychological and somatic phenotypes across adolescence. J Affect Disord. 2018 Apr 1; 230:1-6.

Scott J, Marwaha S, Ratheesh A, Macmillan I, Yung AR, Morriss R, Hickie IB, Bechdolf A. Bipolar At-Risk Criteria: An Examination of Which Clinical Features Have Optimal Utility for Identifying Youth at Risk of Early Transition from Depression to Bipolar Disorders. Schizophr Bull. 2017 Jul 1;43(4):737-744.

Steadman HJ, Silver E, Monahan J, Appelbaum PS, Robbins PC, Mulvey EP, et al. A classification tree approach to the development of actuarial violence risk assessment tools. Law Hum Behav. 2000; 24(1):83-100.

Winton-Brown TT, Harvey SB, McGuire PK. The diagnostic significance of BLIPS (Brief Limited Intermittent Psychotic Symptoms) in psychosis. Schizophr Res. 2011;131(1-3):256-257.

Wright MJ, Martin NG. Brisbane adolescent twin study: Outline of study methods and research projects. Aust J Psychol 2004; 56:65–78.

Yung AR, Phillips LJ, Yuen HP, Francey SM, McFarlane CA, Hallgren M, McGorry PD. Psychosis prediction: 12-month follow up of a high-risk ("prodromal") group. Schizophr Res. 2003 Mar 1;60(1):21-32.

Yung AR, Nelson B, Baker K, Buckby JA, Baksheev G, Cosgrave EM. Psychotic-like experiences in a community sample of adolescents: implications for the continuum model of psychosis and prediction of schizophrenia. Aust N Z J Psychiatry. 2009 Feb;43(2):118-28.
