# Supplementary figures and images for: Early expressions of psychopathology and risk associated with trans-diagnostic transition to mood and psychotic disorders in adolescents and young adults
Source: PLoS One. 2021 Jun 4;16(6):e0252550. doi: 10.1371/journal.pone.0252550 (PMC8177455; doi:10.1371/journal.pone.0252550)

**
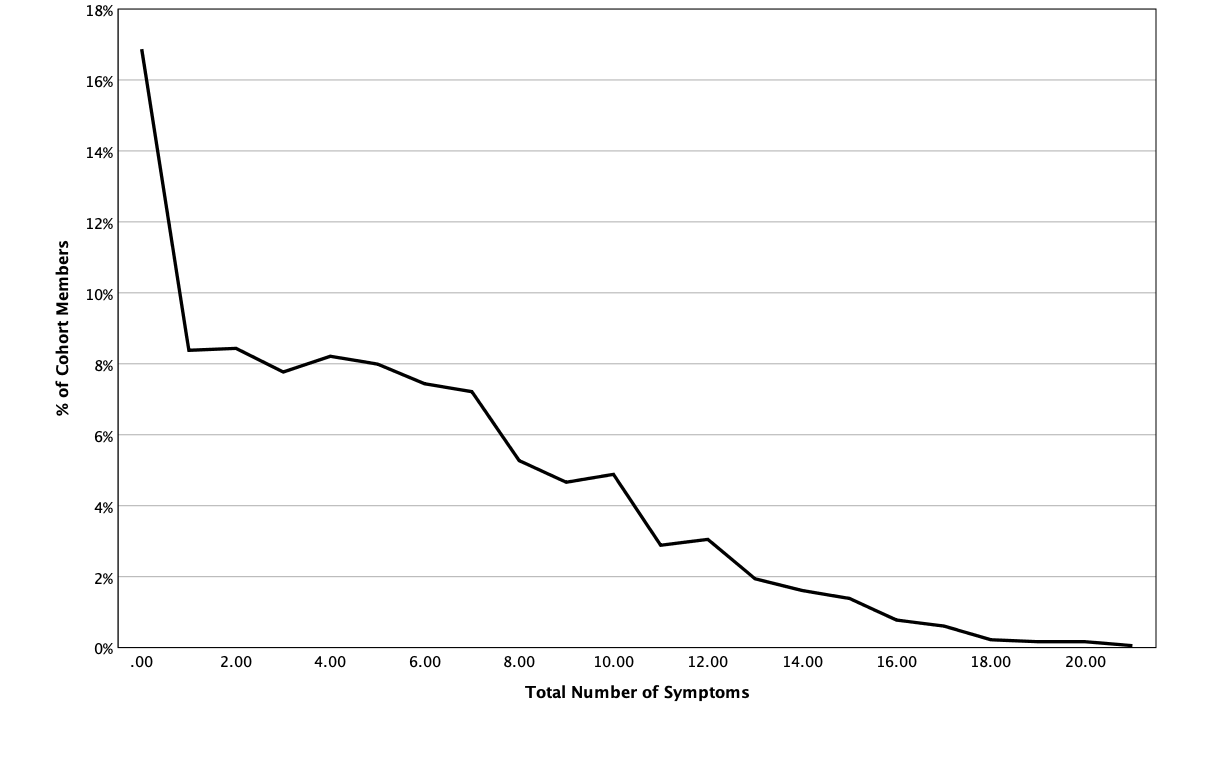
**

**S1 Fig. Proportion of study cohort vs. total symptom score (range 0-23)**

Supplement: S1 Fig — (DOC) [file pone.0252550.s003.doc]
