# Supplementary material for: Early expressions of psychopathology and risk associated with trans-diagnostic transition to mood and psychotic disorders in adolescents and young adults
Source: PLoS One. 2021 Jun 4;16(6):e0252550. doi: 10.1371/journal.pone.0252550 (PMC8177455; doi:10.1371/journal.pone.0252550)
Supplement: S1 Table — (DOC) [file pone.0252550.s004.doc]

| **S1 Table. Comparison of cases included in the study cohort (n=1815) with excluded cases (n=725).** | | | |
| --- | --- | --- | --- |
| **Characteristic** | **Current Study Cohort**  **(N = 1815)** | **Excluded from Study**  **Cohort**  **(N=725)** | **X^2^ or t-test***  **signif. (p)** |
| Mean Age in years (with SD) | 26.4 (4.2) | 26.9 (4.7) | 0.03 |
|  | ***Number (%)*** | ***Number (%)*** |  |
| Females | 1050 (58%) | 413 (57%) | 0.72 |
| Educational Level: Junior or Senior School only | 327 (18%) | 116 (16%) |  |
| Full-Time Employment | 1089 (60%) | 421 (58%) | 0.39 |
| Civil Status: Single | 998 (55%) | 370 (51%) | 0.07 |
| Zygosity: |  |  |  |
| Monozygotic Twins | 509 (28%) | 218 (30%) |  |
| Dizygotic Twins | 698 (38%) | 282 (39%) | 0.42 |
| Non-Twin Siblings | 608 (33%) | 225 (31%) |  |
