# Supplementary material for: Early expressions of psychopathology and risk associated with trans-diagnostic transition to mood and psychotic disorders in adolescents and young adults
Source: PLoS One. 2021 Jun 4;16(6):e0252550. doi: 10.1371/journal.pone.0252550 (PMC8177455; doi:10.1371/journal.pone.0252550)
Supplement: S2 Table — (DOC) [file pone.0252550.s005.doc]

| **S2 Table. Prevalence of individual items** | | | |
| --- | --- | --- | --- |
| **Item Number** | **Item description** | **Prevalence**  **(N = 1815)** | **Percentage**  **(%)*** |
|  | **Depressive Symptoms** |  |  |
| DLE1 | Nervous/Tense | 403 | 22 |
| DLE2 | Sadness/Depressed Mood | 325 | 18 |
| DLE3 | Feel Stressed | 565 | 31 |
| DLE4 | Feel Overwhelmed | 510 | 28 |
| DLE5 | Loss of Confidence | 376 | 21 |
| DLE6 | Hopelessness | 474 | 26 |
| DLE7 | Somatic Pain | 248 | 14 |
| DLE8 | Hypersomnia | 930 | 50 |
| DLE9 | Fatigue | 430 | 24 |
| DLE10 | Impaired Sleep (Quality) | 724 | 40 |
| DLE11 | Impaired Concentration | 466 | 26 |
| DLE12 | Anergia | 534 | 29 |
|  |  |  |  |
|  | **Hypo/Manic Symptoms** |  |  |
| HMLE1 | Feeling Elated | 832 | 46 |
| HMLE2 | Increased Self-Esteem | 710 | 39 |
| HMLE3 | Need Less Sleep | 438 | 24 |
| HMLE4 | Increased Psychomotor Speed (Speech) | 483 | 27 |
| HMLE5 | Increased Activity (Physical) | 607 | 33 |
|  |  |  |  |
|  | **Psychotic Symptoms** |  |  |
| PLE1 | Thoughts Not Your Own | 120 | 7 |
| PLE2 | Third Party Auditory Hallucinations | 20 | 1 |
| PLE3 | Hearing Voices (when alone) | 55 | 3 |
| PLE4 | Feeling Threatened by Others | 64 | 4 |
| PLE5 | Thinking People are Against You (Paranoia) | 79 | 5 |
| PLE6 | Thought Withdrawal | 26 | 1 |
| *Percentages reported to nearest whole number; N= Number.  HMLE: Hypomanic-Like Experiences; DLE: Depression-Like Experiences;  PLE: Psychotic-Like Experiences. | | | |
