# Supplementary material for: Early expressions of psychopathology and risk associated with trans-diagnostic transition to mood and psychotic disorders in adolescents and young adults
Source: PLoS One. 2021 Jun 4;16(6):e0252550. doi: 10.1371/journal.pone.0252550 (PMC8177455; doi:10.1371/journal.pone.0252550)
Supplement: S3 Table — (DOC) [file pone.0252550.s006.doc]

| **S3 Table. CIDI diagnoses, Sub-Threshold syndromes, Family History and Symptom Load for the Study Cohort** | | | | | | | |
| --- | --- | --- | --- | --- | --- | --- | --- |
| **CIDI** | **SubT** | **Sx Load** | | **No FH** | **FH+** | **MULTI-FH** | **Total** |
| **None** | None | Sx_Load | <5 Sx | 564 | 158 | 17 | 739 |
|  |  |  | >=5 Sx | 144 | 66 | 4 | 214 |
|  | 1 | Sx_Load | <5 Sx | 1 | 1 | 0 | 2 |
|  |  |  | >=5 Sx | 181 | 61 | 9 | 251 |
|  | >=2 | Sx_Load | >=5 Sx | 29 | 6 | 2 | 37 |
|  |  | Total | | 29 | 6 | 2 | 37 |
|  | Total | Sx_Load | <5 Sx | 565 | 159 | 17 | 741 |
|  |  |  | >=5 Sx | 354 | 133 | 15 | 502 |
|  |  | Total (None) | | 919 | 292 | 32 | 1243 |
| **1 Diagnosis** | None | Sx_Load | <5 Sx | 101 | 45 | 3 | 149 |
|  |  |  | >=5 Sx | 56 | 29 | 5 | 90 |
|  | 1 | Sx_Load | <5 Sx | 0 | 1 | 0 | 1 |
|  |  |  | >=5 Sx | 95 | 58 | 11 | 164 |
|  | >=2 | Sx_Load | >=5 Sx | 27 | 10 | 1 | 38 |
|  |  | Total | | 27 | 10 | 1 | 38 |
|  | Total | Sx_Load | <5 Sx | 101 | 46 | 3 | 150 |
|  |  |  | >=5 Sx | 178 | 97 | 17 | 292 |
|  |  | Total (1 Diagnosis) | | 279 | 143 | 20 | 442 |
| **>=2 Diagnoses** | None | Sx_Load | <5 Sx | 12 | 5 | 0 | 17 |
|  |  |  | >=5 Sx | 11 | 12 | 2 | 25 |
|  | 1 | Sx_Load | >=5 Sx | 34 | 15 | 5 | 54 |
|  | >=2 | Sx_Load | >=5 Sx | 16 | 16 | 2 | 34 |
|  | Total | Sx_Load | <5 Sx | 12 | 5 | 0 | 17 |
|  |  |  | >=5 Sx | 61 | 43 | 9 | 113 |
|  |  | Total (>=2 Diagnoses) | | 73 | 48 | 9 | 130 |
| **Total** | None | Sx_Load | <5 Sx | 677 | 208 | 20 | 905 |
|  |  |  | >=5 Sx | 211 | 107 | 11 | 329 |
|  | 1 | Sx_Load | <5 Sx | 1 | 2 | 0 | 3 |
|  |  |  | >=5 Sx | 310 | 134 | 25 | 469 |
|  | >=2 | Sx_Load | >=5 Sx | 72 | 32 | 5 | 109 |
|  |  | Total | | 72 | 32 | 5 | 109 |
|  | Total | Sx_Load | <5 Sx | 678 | 210 | 20 | 908 |
|  |  |  | >=5 Sx | 593 | 273 | 41 | 907 |
|  | **Total (All Subgroups)** | | | **1271** | **483** | **61** | **1815** |
| CIDI: Composite International Diagnostic Interview; Sub T: Subthreshold syndromes; FH; Family History (of unipolar, bipolar and/or psychotic disorder); Multi-FH: family history in >=2 first degree relatives; Sx Load: total number of self-reported symptoms classified as above (>=5) or below (<5) the sample median. | | | | | | | |
